# Axillary Peripheral Nerve Schwannoma: A Rare Occurrence in the Torso

**DOI:** 10.7759/cureus.73622

**Published:** 2024-11-13

**Authors:** Ahmed Inayath Syed, Sachin Patil, Sneha Zakkir

**Affiliations:** 1 Department of General Surgery, Vydehi Institute of Medical Sciences and Research Centre, Bangalore, IND

**Keywords:** axillary schwanomma, benign peripheral nerve sheath tumor, brachial plexus, brachial plexus schwannoma, excisional biopsy

## Abstract

A schwannoma is a benign, solitary, noninvasive, and encapsulated tumor that originates from Schwann cells of the peripheral nerve sheath commonly found in the head and neck. A rare case of a benign schwannoma in the axillary region of a 34-year-old male patient is presented here accompanied by a discussion on the known entities of peripheral nerve schwannoma as well as the clinical and radiological findings coupled with treatment techniques. The patient presented with a left axillary mass of seven years gradually progressing in size which is associated with pain in the left arm. There were no neurological deficits on examination. Ultrasound and soft tissue MRI were performed followed by fine-needle aspiration cytology (FNAC), and a decision was made to take the patient up for an excisional biopsy. Histopathology revealed a tumor composed of hyper- (Antoni A) and hypocellular (Antoni B) areas, while immunohistochemistry was positive for the S-100 protein, thus confirming the diagnosis of left axillary peripheral nerve schwannoma. With such nonspecific presentation and the added challenge of its rarity, an axillary schwannoma may be easily missed and mismanaged. Surgical excision and biopsy are recommended with an aim of preserving neurological function.

## Introduction

A schwannoma is a benign, solitary, noninvasive, and encapsulated tumor that originates from Schwann cells of the peripheral nerve sheath. Schwann cells are the myelinating cells of the peripheral nervous system that play an essential role in their function and maintenance. Though schwannomas usually present as a painful mass associated with sensory and motor deficits, these peripheral nerve tumors have a varied range of nonspecific manifestations. Neurilemmomas, sometimes known as schwannomas, are also in the same category [1-3].

They comprise an important differential for lesions of the head and neck. It is fairly uncommon for a schwannoma to develop in the foot and ankle, but a brachial plexus presentation is seldom heard of with a prevalence rate of 5% compared to that of the former which ranges from 1% to 10%, hence marking it a unique phenomenon that few clinicians come across [4].

Such a case of a benign schwannoma in the axillary region is presented here for your consideration. In addition, the authors discuss and examine the known entities of peripheral nerve schwannoma and present the clinical and radiological findings alongside the treatment techniques for the purpose of accurately diagnosing and managing a peripheral nerve schwannoma.

## Case presentation

This is a 34-year-old male patient with no known comorbidities who presented to the General Surgery outpatient department with a swelling in the left axillary region for seven years which was insidious in onset and gradually progressive. Initially, it measured 2 x 2 cm approximately and has since doubled in size. The patient also gives a history of pricking pain in the left arm that is nonradiating, aggravates during winter and on exertion, and is spontaneously relieved with rest. Along with these symptoms, the patient also reported a history of a tingling sensation and numbness in the tips of his fingers and toes, which is worse during winter. The patient reported no episodes of fever, trauma, or a loss of weight or appetite. He had no relevant past medical or surgical history. On enquiring further, we did not find any plausible family history to guide the current diagnosis.

On examination, the patient appeared to be of moderate build and well-nourished, with stable hemodynamics. The systemic examination was found to be grossly normal. Following this, on local examination, we noted a solitary spherical swelling of size 4 x 4 cm over the left axillary region which was ovoid with a smooth surface and well-defined margins. The skin over the swelling had no obvious dilated vessels or scars. When palpated, we observed that the swelling was nontender and firm in consistency, with restricted mobility in the horizontal plane. When the latissimus dorsi muscle was contracted, the swelling appeared to be more prominent. Additionally, a detailed neurological examination was performed, which revealed no obvious sensory and motor deficits. Hoffmann-Tinel sign was noted to be negative.

At this stage, a differential diagnosis of lipoma, lymph node conglomerate, nerve sheath tumor, and soft tissue tumor was made. To isolate the diagnosis, the patient underwent imaging and biopsy.

**Figure 1:**
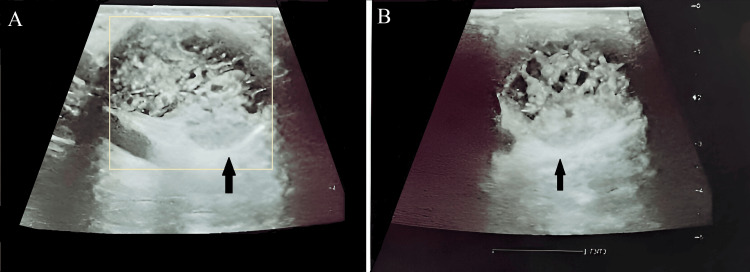
Ultrasound images of the left axilla showing a well-defined, heterogeneous cystic solid lesion with brachial vessels, measuring 37 x 33 x 29 mm (shown in black arrow)

Ultrasonography of the left axilla (Figures 1A-1B) revealed a 37 x 33 x 29 mm, well-defined, heterogeneous cystic solid lesion with brachial vessels. There was evidence of increased vascularity in the mass. However, there was no obvious lymph node mass.

**Figure 2:**
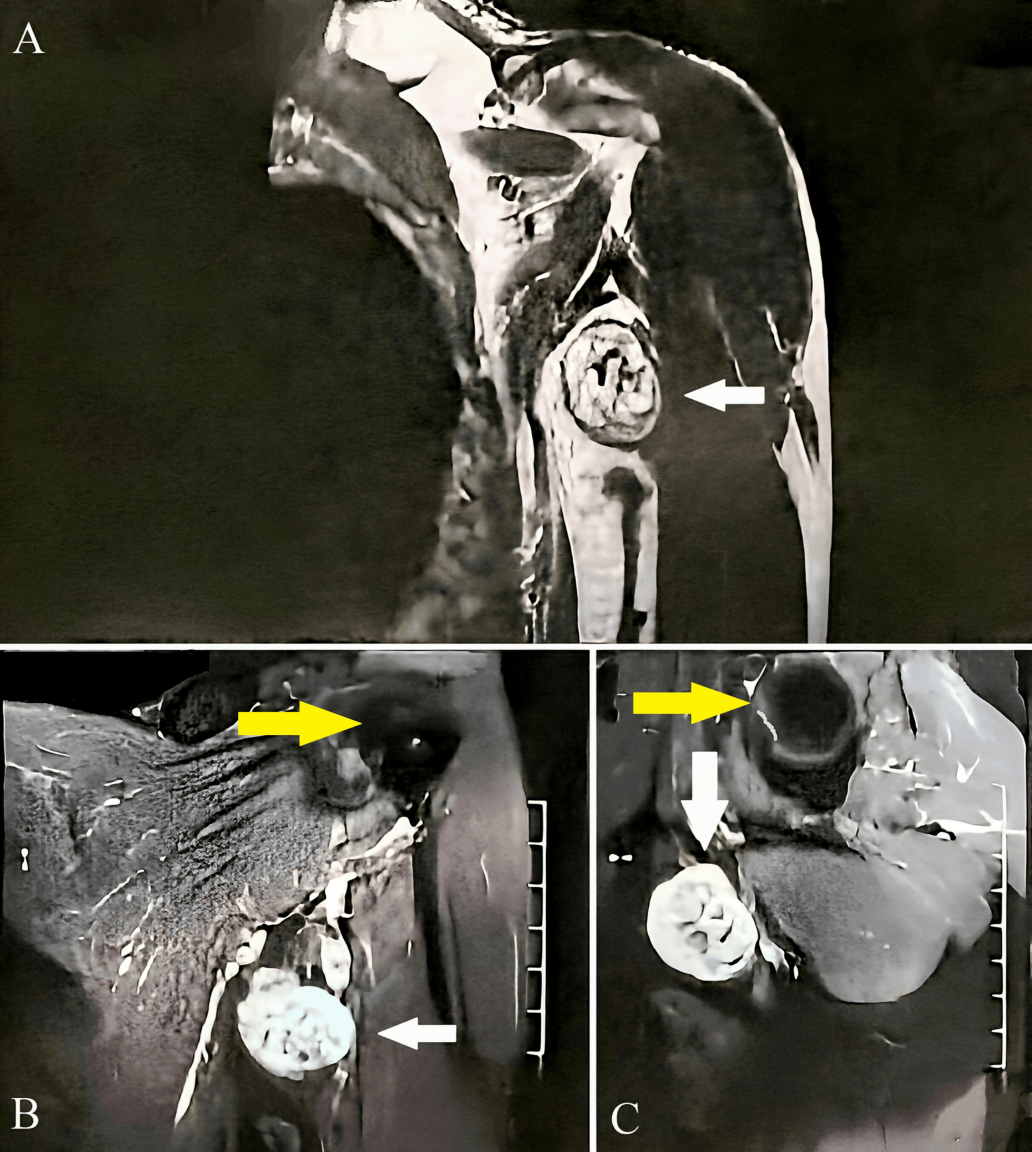
Contrast-enhanced magnetic resonance images of the left shoulder showing a well-encapsulated T1 hypointense, T2 isointense, and STIR hyperintense mass lesion (shown with a white arrow) with heterogeneous enhancement on contrast study involving anteroinferior aspect of the left axilla region inferior to the axillary vessels in coronal view (A on the top and B on the bottom left) and axial view (C on the bottom right). Head of the humerus shown with a yellow arrow STIR: Short-TI inversion recovery

A contrast-enhanced MRI of the left shoulder (Figures 2A-2C) showed a well-encapsulated T1 hypointense, T2 isointense, and short-TI inversion recovery (STIR) hyper-intense mass lesion showing heterogeneous enhancement on contrast study involving antero-inferior aspect of the left axilla region inferior to the axillary vessels, likely representing a nerve sheath tumor.

**Figure 3:**
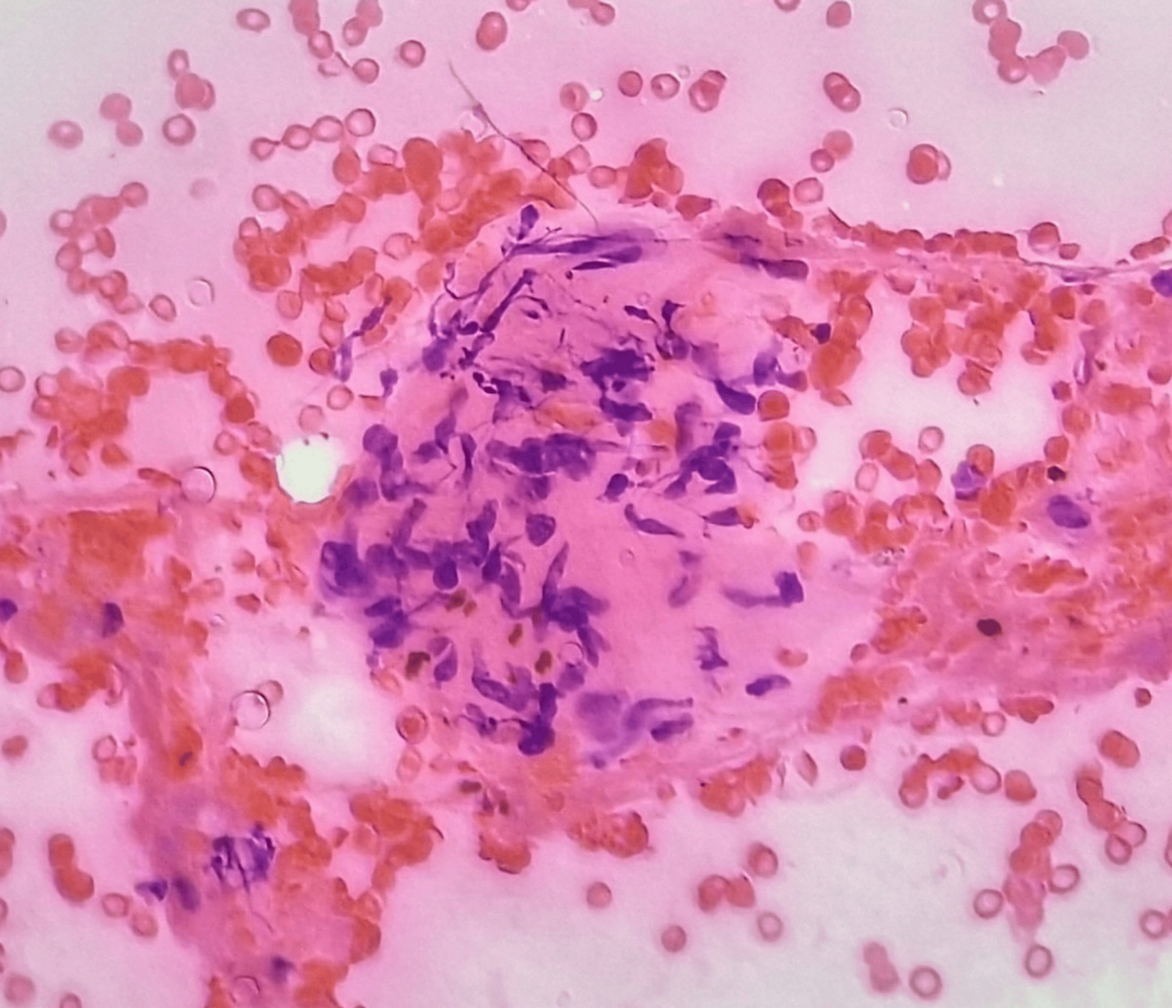
Microphotograph of fine-needle aspiration cytology of the swelling showing loosely cohesive spindle cell fragments having bland spinal nuclei with pointed ends and indistinct cell borders, in a background of fibrillary material (H and E, x40)

Fine-needle aspiration cytology (FNAC) (Figure 3) reported features suggestive of benign spindle cell neoplasm, possibly neural in origin.

**Figure 4:**
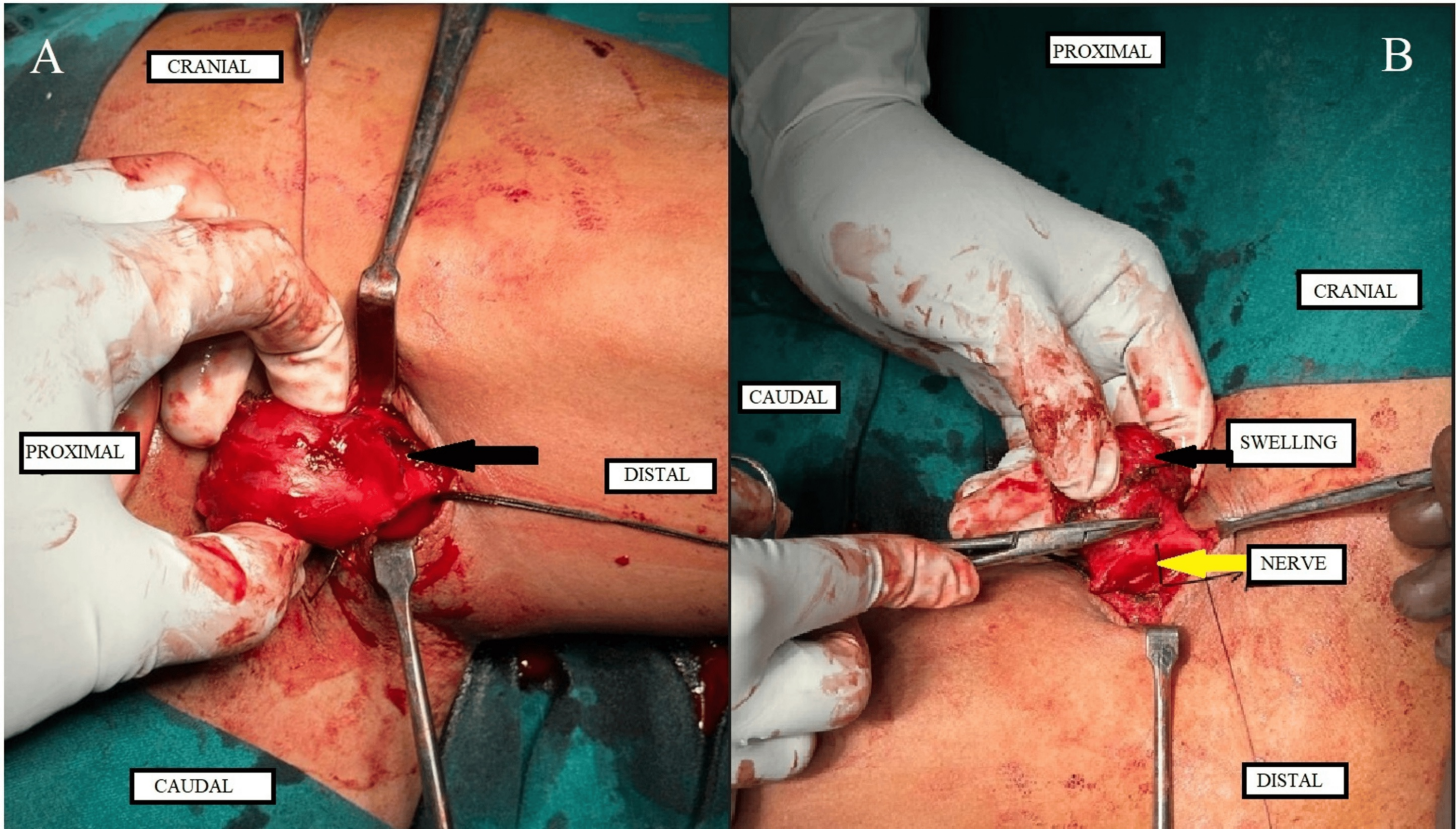
Operative images from the excision biopsy (A on the left and B on the right) showing the mass (marked with a black arrow). Image on the right (B) shows adherent neurovasculature-cords or branches of the brachial plexus (marked with a yellow arrow)

The patient then underwent an excisional biopsy (Figures 4A-4B). The mass was found to be above the plane of the muscle and adherent to the underlying neurovasculature. A complete excision of the mass was performed. Examination of the axillary lymph nodes did not yield any significant lymphadenopathy.

**Figure 5:**
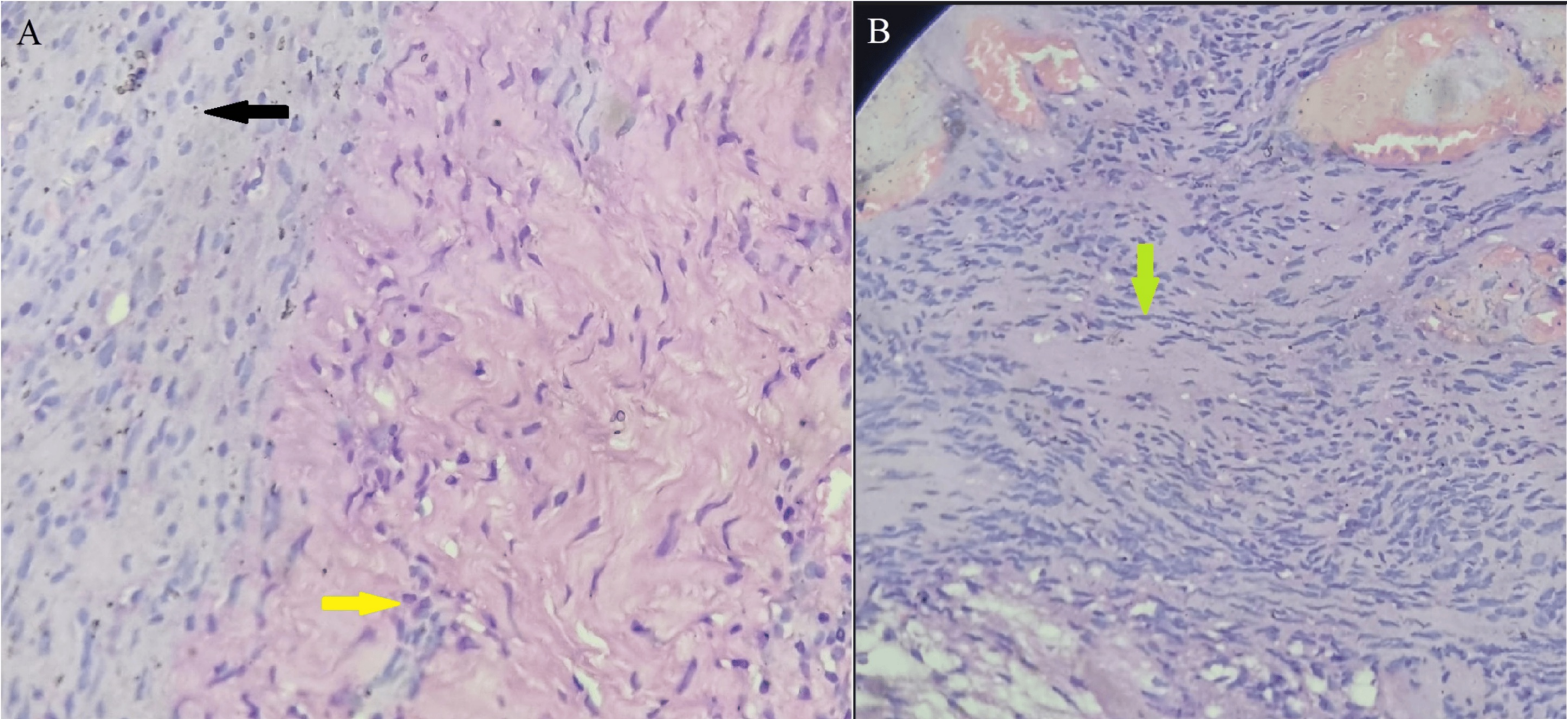
On the left (A) is a histopathological image of the excised mass showing a hypercellular area (Antoni A) with fascicular pattern of uniform spindle cells (shown in black arrow) and a hypocellular area (Antoni B) with myxoid character and occasional Verocay bodies (shown in yellow arrow). On the right (B) is a histopathological image showing a Verocay body (shown in green arrow) (H and E, x40)

Histopathology revealed a tumor composed of hyper- and hypocellular areas (Figures 5A-5B). The hypercellular area (Antoni A) was formed by uniform spindle cells arranged in fascicular pattern. These cells had ill-defined cytoplasm and bland elongated nuclei with dense chromatin. The hypocellular area (Antoni B) was formed by myxoid areas. Areas of hyalinization were noted, and occasional Verocay bodies were seen. Many hyalinized blood vessels with perivascular hemosiderin were noted with areas of hemorrhage. Additionally, areas were seen with dense aggregates of lymphocytes, plasma cells, and many foamy macrophages. ​​​​​These features were consistent with peripheral nerve schwannoma.

**Figure 6:**
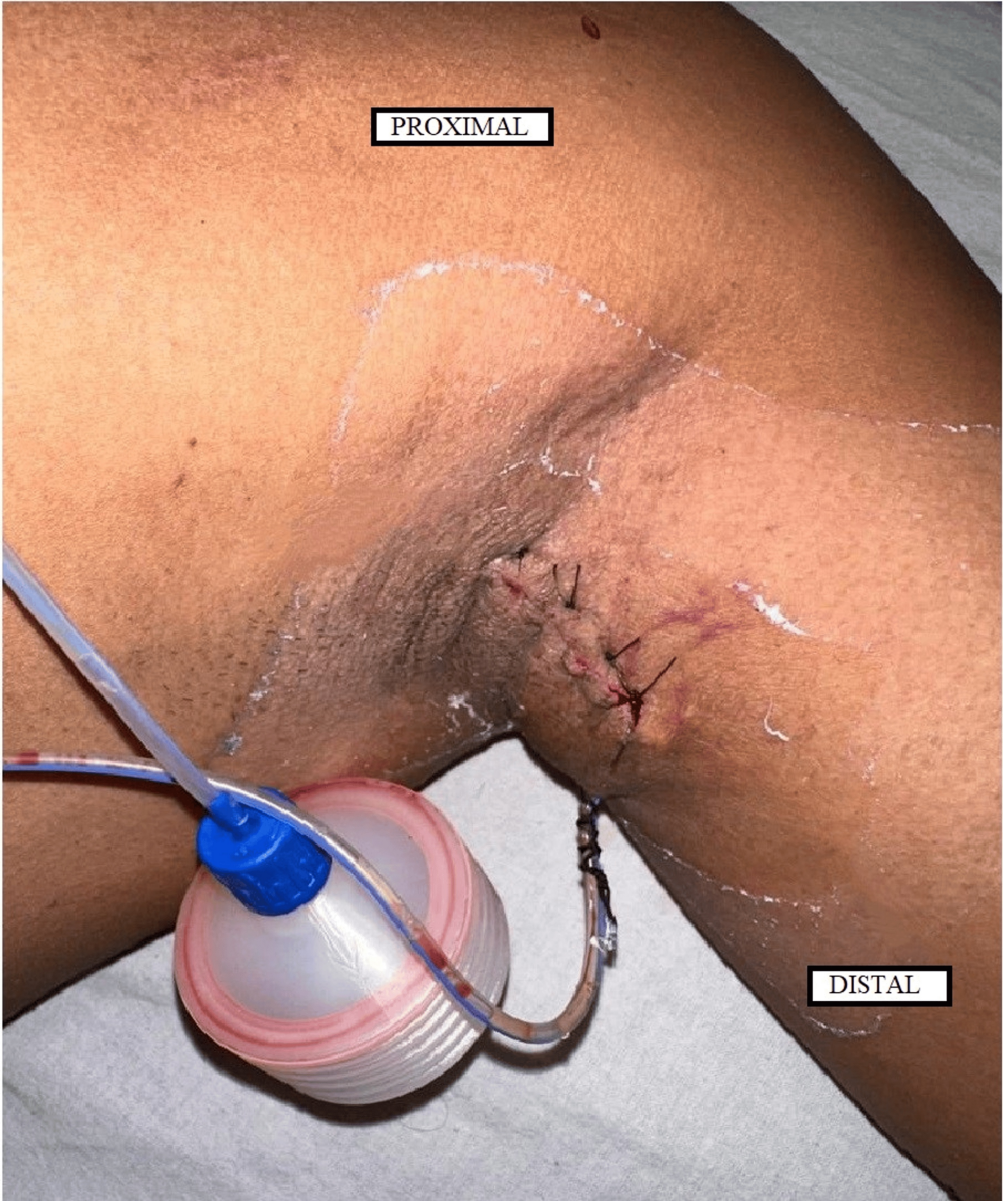
Image of the excision site on postop day two with surgical drain in situ showing a 4 cm long sutured incision and minimal fluid collected in the drain

Immunohistochemistry was positive for the S-100 protein. The postoperative period (Figure 6) was uneventful, and the surgical drain was removed on postop day two. The patient was discharged on postop day four and is on regular follow-up since the surgery with no complaints of any neurological deficits or recurrence.

## Discussion

Schwannoma is a solitary, firm, well‑circumscribed, and encapsulated peripheral nerve sheath tumor [1-3]. It is commonly round or ovoid with a slow-growing and non-infiltrating nature, reaching a size ranging from 1.5 cm to 3 cm in diameter [5]. Schwannomas do not discriminate based on gender, and they are most frequently found in people who are in their fourth decade of life [3]. Schwannomas are benign but extremely rarely can undergo malignant transformation which may occur in association with schwannomatosis, neurofibromatosis, or following therapeutic irradiation [6].

Though schwannomas are the most common peripheral nerve tumors, this diagnosis enters into the picture only when the mass is rooted in the head and neck region and is often neglected in differentials for other anatomical swellings due to its infrequency. But similar to the case we have highlighted, it can arise in the torso as a tumor originating from the cords or branches of the brachial plexus subsequently manifesting in the axilla as an atypical expression accounting for only 5% of all schwannomas [3,7].

Clinically, it can cause either an asymptomatic or a painful lump with or without neurological deficits [2,8]. Local swelling, motor impairments, hypoesthesia, and neuropathic pain might result from pressure on unaffected nerve fascicles or loss of function of the affected fascicles. These symptoms may be misconstrued as radicular symptoms or other common illnesses including undiagnosed joint and muscle pain due to their nonspecific character [2,8,9].

A rigorous physical examination is pivotal to the assessment of patients with brachial plexus tumors as the initial symptoms and signs are few and frail [8]. A local swelling or palpable lump may lead doctors to misinterpret the tumor origin without an appropriate neurological examination. The differential then follows the beaten path of pathological lymph nodes, metastasis, paraganglioma, non-nervous soft tissue, or vascular tumors [10].

Thus, diagnostic tests include neurological exams, MRI, ultrasound, and electrophysiology [11]. High-resolution nerve sonography for peripheral neuropathies is becoming more widespread [12]. Although there are quite a few modalities in modern medicine that greatly aid in the detection of such anomalies, according to the findings of a retrospective study done on the diagnosis of benign peripheral nerve sheath tumors (bPNST) by Uerschels et al. in 2021, it was noted that spinal MRI did not prevent misdiagnosis because 76% of patients had unneeded or insufficient therapy. A CT-guided biopsy can damage nerves and is always a topic of dispute between professionals. Often, bPNSTs of the brachial plexus were misdiagnosed as malignant lymph nodes or soft tissue sarcoma. In those cases, anatomical relation to the plexus was not detected or misinterpreted; therefore, bPNST as a possibility was overlooked [13]. FNA is one of the methods of diagnosing schwannoma, although it is difficult to recognize tissue architectural pattern on cytology [14]. Upon searching through literature, only a single case of axillary schwannoma has been reported which was diagnosed preoperatively by ultrasound-guided tru-cut needle biopsy with no complications [15].

The previously referenced study also strongly suggests that misdiagnosis leads to mistreatment as 26.3% of patients underwent an unnecessary surgical intervention unrelated to the actual lesion, including cervical spine exploration, dental surgery, and shoulder arthroscopy. This caused serious neurological impairments that required revision surgery to remove tumor tissue or perform neurolysis [13].

The gold standard treatment for schwannomas is complete surgical excision as a definitive course while ensuring no function loss. This should be the primary aim, especially if these benign tumors have not caused neurological impairments [16]. Complete subcapsular schwannoma excision seldom causes recurrence [17]. The use of intraoperative nerve action potential devices has been reported to be on the rise to minimize any collateral nerve damage [18].

Most symptomatic patients receive definitive diagnosis and proper treatment late. Failure to diagnose a nerve tumor as the cause of pain or sensation deficiencies might delay treatment or lead to, chronic pain syndrome, or permanent neurological deficits which can be psychologically taxing and have lifelong implications [19].

## Conclusions

This is our case report of an axillary peripheral nerve schwannoma that we have put forth to consider its inclusion in the differential diagnosis of swellings of the axillary region. They are often missed due to the challenging nature of their presentation; however, evidence shows that this must be kept in mind while planning any surgical intervention, and because it is an infrequent occurrence, misdiagnosis and subsequent mistreatment can have a significant long-standing neurological impact. Timely detection and surgical management have been found to lead to an excellent short-term and long-term prognosis.
